# Carotid intima-media thickness and arterial stiffness in relation to cerebral small vessel disease in neurologically asymptomatic individuals with type 1 diabetes

**DOI:** 10.1007/s00592-021-01678-x

**Published:** 2021-03-20

**Authors:** Jussi Inkeri, Anniina Tynjälä, Carol Forsblom, Ron Liebkind, Turgut Tatlisumak, Lena M. Thorn, Per-Henrik Groop, Sara Shams, Jukka Putaala, Juha Martola, Daniel Gordin, FinnDiane Study Group

**Affiliations:** 1 HUS Medical Imaging Center, Radiology, University of Helsinki and Helsinki University Hospital, Helsinki, Finland; 2 Folkhälsan Institute of Genetics, Folkhälsan Research Center, Helsinki, Finland; 3 Abdominal Center, Nephrology, University of Helsinki and Helsinki University Hospital, Helsinki, Finland; 4 Research Program for Clinical and Molecular Metabolism, University of Helsinki, Helsinki, Finland; 5 Neurology, University of Helsinki and Helsinki University Hospital, Helsinki, Finland; 6 Department of Clinical Neuroscience/Neurology, Institute of Neuroscience and Physiology, Sahlgrenska Academy, University of Gothenburg, Gothenburg, Sweden; 7 Department of Neurology, Sahlgrenska University Hospital, Gothenburg, Sweden; 8 Department of General Practice and Primary Health Care, University of Helsinki and Helsinki University Hospital, Helsinki, Finland; 9 Department of Diabetes, Central Clinical School, Monash University, Melbourne, Australia; 10 Department of Radiology, Karolinska University Hospital, Stockholm, Sweden; 11 Department of Clinical Neuroscience, Karolinska Institute, Stockholm, Sweden; 12 Department of Radiology, Stanford University, Stanford, CA USA; 13 Joslin Diabetes Center, Harvard Medical School, Boston, MA USA

**Keywords:** Carotid intima-media thickness, Arterial tonometry, Cerebral microbleed, Magnetic resonance imaging

## Abstract

**Aims:**

To determine if arterial functional and structural changes are associated with underlying cerebral small vessel disease in neurologically asymptomatic individuals with type 1 diabetes.

**Methods:**

We enrolled 186 individuals (47.8% men; median age 40.0, IQR 33.0—45.0 years) with type 1 diabetes (median diabetes duration of 21.6, IQR 18.2—30.3 years), and 30 age- and sex-matched healthy controls, as part of the Finnish Diabetic Nephropathy (FinnDiane) Study. All individuals underwent a biochemical work-up, brain magnetic resonance imaging (MRI), ultrasound of the common carotid arteries and arterial tonometry. Arterial structural and functional parameters were assessed by carotid intima-media thickness (CIMT), pulse wave velocity and augmentation index.

**Results:**

Cerebral microbleeds (CMBs) were present in 23.7% and white matter hyperintensities (WMHs) in 16.7% of individuals with type 1 diabetes. Those with type 1 diabetes and CMBs had higher median (IQR) CIMT 583 (525 – 663) μm than those without 556 (502 – 607) μm, *p* = 0.016). Higher CIMT was associated with the presence of CMBs (*p* = 0.046) independent of age, eGFR, ApoB, systolic blood pressure, albuminuria, history of retinal photocoagulation and HbA_1c_. Arterial stiffness and CIMT were increased in individuals with type 1 diabetes and WMHs compared to those without; however, these results were not independent of cardiovascular risk factors.

**Conclusions:**

Structural, but not functional, arterial changes are associated with underlying CMBs in asymptomatic individuals with type 1 diabetes.

**Supplementary Information:**

The online version contains supplementary material available at (10.1007/s00592-021-01678-x).

## Introduction

Cerebrovascular disease is a common, albeit scarcely studied, vascular complication in individuals with type 1 diabetes (T1D), who have a fourfold risk of stroke compared to non-diabetic individuals [1]. Although cerebrovascular disease in general is considered to mostly affect large arteries supplying the brain, recent data indicate that, in fact, the small cerebral vessels are particularly affected in type 1 diabetes [2]. Cerebral small vessel disease (cSVD) involves the arterioles, capillaries and venules, with the two most common underlying etiologies being cerebral amyloid angiopathy and hypertensive vasculopathy. Both these etiologies lead to frail microvasculature and an increased risk of intracerebral hemorrhage (ICH). Since the cerebral microvasculature is too small to study per se with neuroimaging, surrogate markers of small vessel disease are employed instead. Magnetic resonance imaging (MRI) findings of cSVD include cerebral microbleeds (CMBs), white matter hyperintensities (WMHs) and lacunar infarcts [3]. We recently showed that cSVD, and especially CMBs, are more common in individuals with type 1 diabetes than in healthy controls [2]. Of note, cSVD has been shown to have a significant impact in type 1 diabetes, as WMHs are associated with cognitive difficulties in these individuals [4], and CMBs are associated with increased risk of ischemic and hemorrhagic strokes [5, 6] as well as premature mortality in the general population [7].

Structural and functional measures of the cerebrovascular system can give insight into the underlying disease burden and the risk of future events. Measurements such as carotid intima-media thickness (CIMT), pulse wave velocity (PVW) and central augmentation index (AIx) all reflect potential vascular pathology, and their relationship with cSVD in individuals with type 1 diabetes is unknown.

The aim of this study was to determine whether arterial functional and structural changes are associated with cSVD in neurologically asymptomatic individuals with type 1 diabetes.

## Methods

### Demographics

This study was performed as part of the Finnish Diabetic Nephropathy (FinnDiane) Study, a nationwide multicenter study aiming to identify genetic, environmental and clinical risk factors for micro- and macrovascular complications in type 1 diabetes [2]. A total of 191 individuals with type 1 diabetes with an age span ranging from 18 to 50 years and with an onset of diabetes < 40 years were included. Exclusion criteria were presence of end-stage renal disease, any clinical signs of cerebrovascular disease, as well as contraindications for MRI. Five individuals with type 1 diabetes were excluded due to missing carotid ultrasound data. Thus, a total of 186 individuals with type 1 diabetes were included in the present study. In addition, 30 healthy age- and sex-matched non-diabetic control subjects were recruited. The study was carried out in accordance with the Declaration of Helsinki and approved by the Ethics Committee of the Helsinki and Uusimaa Hospital District. Each participant signed a written informed consent. [2]

All participants were studied at the FinnDiane Research Center (Biomedicum Helsinki) and the Medical Imaging Center at the Helsinki University Hospital. All underwent brain MRI scans, carotid ultrasound, arterial tonometry, blood tests and a thorough clinical examination.

Blood samples were collected to analyze serum creatinine concentrations, serum lipids and lipoproteins (total cholesterol, high-density lipoprotein [HDL], triglycerides and apolipoprotein B [ApoB]), high-sensitivity C-reactive protein (hsCRP) and blood glycated hemoglobin (HbA_1c_). The Friedewald equation was used to calculate the low-density lipoprotein (LDL) concentration [8], and the CKD-EPI-formula was used to calculate the estimated glomerular filtration rate (eGFR) [9]. Hypertension was defined as an office measurement of systolic blood pressure (SBP) ≥ 140, diastolic blood pressure (DBP) ≥ 90 or the use of anti-hypertensive medication. Obesity was defined as body mass index (BMI) ≥ 30 kg/m^2^. Smoking was defined as a history of smoking. Diabetic kidney disease was defined by the urinary albumin excretion rate (UAER) in two out of three timed urine collections as microalbuminuria (UAER ≥ 20 < 200 μg) or macroalbuminuria (UAER ≥ 200 μg). The presence of retinopathy was divided into three categories: no retinopathy, presence of any retinopathy and history of retinal photocoagulation. In-depth questionnaires, which have been described previously, were utilized in the collection of clinical data [10]. Of the individuals with type 1 diabetes, two had missing data regarding smoking, five regarding diabetic kidney disease and three regarding retinopathy.

### Imaging

Brain MRI was performed with a 3.0 T scanner (Achieva; Philips, Best, the Netherlands) and included T1, T2, FLAIR, SWI, T2*, DWI, T1 MPRAGE and 3D TOF sequences. Brain MRI images were assessed by an experienced neuroradiologist (JM), who was blinded to all clinical data. Markers of cSVD were rated per the standardized STRIVE criteria, including the assessment of CMBs, WMHs (Fazekas scale used, with category ≥ 1 considered a significant burden) and lacunar infarcts [11].

### Structural and functional arterial measurements

Carotid ultrasound was performed on the left and right carotid arteries. The distal 1 cm segment of the common carotid artery segment, immediately before the origin of the bulb was scanned using an ultrasound scanner equipped with a 10 MHz linear probe (MyLab 70, Esaote, Genova, Italy) and implemented with a radiofrequency-based tracking of arterial wall (QIMT®) that allows an automatic and real-time determination of far-wall CIMT [12, 13]. The mean of two measurements of the left and right CIMT was calculated for the subsequent analyses. Arterial stiffness was estimated by applanation tonometry (SphygmoCor, Atcor Medical, Sydney, NSW, Australia) using a high-fidelity micromanometer (SPC-301; Millar Instruments, Houston, TX) [14]. Pressure waveforms were recorded sequentially at the carotid, femoral and radial artery, with the participant in the supine position and the R wave of a simultaneous electrocardiography (ECG) recording as reference frame. To calculate central pulse wave velocity (cPWV) and brachial pulse wave velocity (bPWV), the path lengths from the sternal notch to the femoral and radial palpable pulses, respectively, were measured. In addition, three sequential recordings with a minimum of 20 pressure waveforms were recorded from the right radial artery for pulse wave analysis. AIx was calculated from a central pressure waveform, generated with a standardized transfer function, as the ratio of the heart rate adjusted augmentation pressure (difference between the second and the first systolic peak) and the central pulse pressure. AIx data were missing from eight individuals, bPWV data from 19 and cPWV data from 30 individuals with type 1 diabetes.

### Statistics

Statistical analysis was done with IBM SPSS Statistics 25.0 (IBM, Armonk, NY).* T* tests were used for the parametric data presented as means (±SD), and Mann–Whitney U and Kruskal–Wallis tests for the nonparametric data presented as medians (interquartile range). The *X*^2^ test or Fisher’s exact tests were performed for categorical variables. Individuals with type 1 diabetes were subdivided into three groups based on the number of CMBs (zero, one to two, more than two) [15]. Bivariate (Pearson) correlation analysis was used to study correlations between CIMT, arterial stiffness and the number of CMBs and WMHs. Multivariable logistic regression models were built with the arterial parameters (CIMT, bPWV, cPWV and AIx) as independent variables and markers of cSVD as dependent variables and adjusted for clinically relevant risk factors contributing to cerebrovascular disease (age, sex, diabetes duration, BMI, albuminuria, history of retinal photocoagulation, HbA_1c_, eGFR, LDL, ApoB, systolic blood pressure, statin therapy and antihypertensive medication). The threshold for statistical significance was set at *p* < 0.05.

## Results

### Clinical characteristics

A total of 186 individuals with type 1 diabetes and 30 healthy age- and sex-matched non-diabetic control subjects were enrolled and met the inclusion criteria for this study, with demographics previously presented [2]. Briefly, the individuals with type 1 diabetes had higher BMI, systolic blood pressure and HbA_1c_, as well as higher hsCRP. The prevalence of cSVD and CMBs was higher in individuals with type 1 diabetes compared to healthy controls. Of the 186 individuals with type 1 diabetes, 64 (34.4%) had signs of cSVD, 44 (23.7%) had CMBs, 31 (16.7%) WMHs and 4 (2.2%) lacunar infarcts (Table 1).

**Table 1:** The clinical characteristics, prevalence of cSVD and characteristics of arterial structure and function in individuals with type 1 diabetes vs. healthy controls

	Individuals with type 1 diabetes *N* = 186	Controls *N* = 30	*p*
Age, years, median (IQR)	40.0 (33.0–45.0)	38.4 (31.4–43.2)	0.475
Male sex, n (%)	89 (47.8)	13 (56.7)	0.646
Diabetes duration, years, median (IQR)	21.6 (18.2–30.3)	0	-
Body mass index, kg/m^2^, median (IQR)	26.2 (24.0–28.7)	24.3 (22.5–25.5)	0.004
Systolic blood pressure, mmHg, median (IQR)	129.3 (119.0–139.5)	121.0 (116.0–128.0)	0.003
HbA_1c_, %, median (IQR)	8.1 (7.4–8.9)	5.2 (4.9–5.4)	< 0.001
Total cholesterol, mmol/L, median (IQR)	4.4 (4.0–4.9)	4.4 (4.2–5.5)	0.243
Low-density lipoprotein, mmol/L, median (IQR)	2.4 (2.1–3.0)	2.6 (2.1–3.2)	0.156
Triglycerides, mmol/L, median (IQR)	0.9 (0.7–1.4)	0.8 (0.7–1.4)	0.640
Apolipoprotein B, g/L, median (IQR)	67.0 (59.0–83.0)	69.0 (57.3–94.5)	0.491
High-sensitivity C-reactive protein, mg/L, median (IQR)	1.2 (0.5–2.9)	0.4 (0.2–1.5)	0.002
cSVD, n (%)	64 (34.4)	3 (10)	0.007
CMBs, n (%)	44 (23.7)	1 (3.3)	0.011
WMHs, n (%)	31 (16.7)	2 (6.7)	0.271
Lacunae, n (%)	4 (2.2)	0 (0)	1.000
CIMT, μm, median (IQR)	566 (507–622)	491 (458–554)	< 0.001
Central PWV, m/s, median (IQR)	7.3 (6.1–8.8)	6.7 (5.3–7.2)	0.003
Brachial PWV, m/s, mean ± SD	7.6 ± 1.3	7.8 ± 1.1	0.363
AIx, %, median (IQR)	14.9 (6.6–22.0)	11.5 (5.8–19.2)	0.140

IQR = interquartile range, CIMT = carotid intima-media thickness, PWV = pulse wave velocity, AIx = augmentation index, cSVD = cerebral small-vessel disease, CMBs = cerebral microbleeds, WMHs = white matter hyperintensities

### CIMT and arterial stiffness in individuals with T1D and healthy control subjects

CIMT (median [IQR]) (566 [507 – 622] μm vs. 491 [458 – 554] μm, *p* < 0.001) and cPWV (7.3 [6.1—8.8] m/s vs. 6.7 [5.3—7.2] m/s, *p* = 0.003) were higher in individuals with type 1 diabetes compared to healthy controls. No differences in AIx (14.9% [6.6—22.0] vs. 11.5% [5.8 – 19.2], *p* = 0.363) or bPWV (7.6 m/s ± 1.3 vs. 7.8 ± 1.1 m/s, *p* = 0.140) were observed between the groups. Further, comparisons of cSVD, CIMT and arterial stiffness parameters between the studied groups are presented in Table 1.

### CIMT and cSVD

**Fig. 1:**
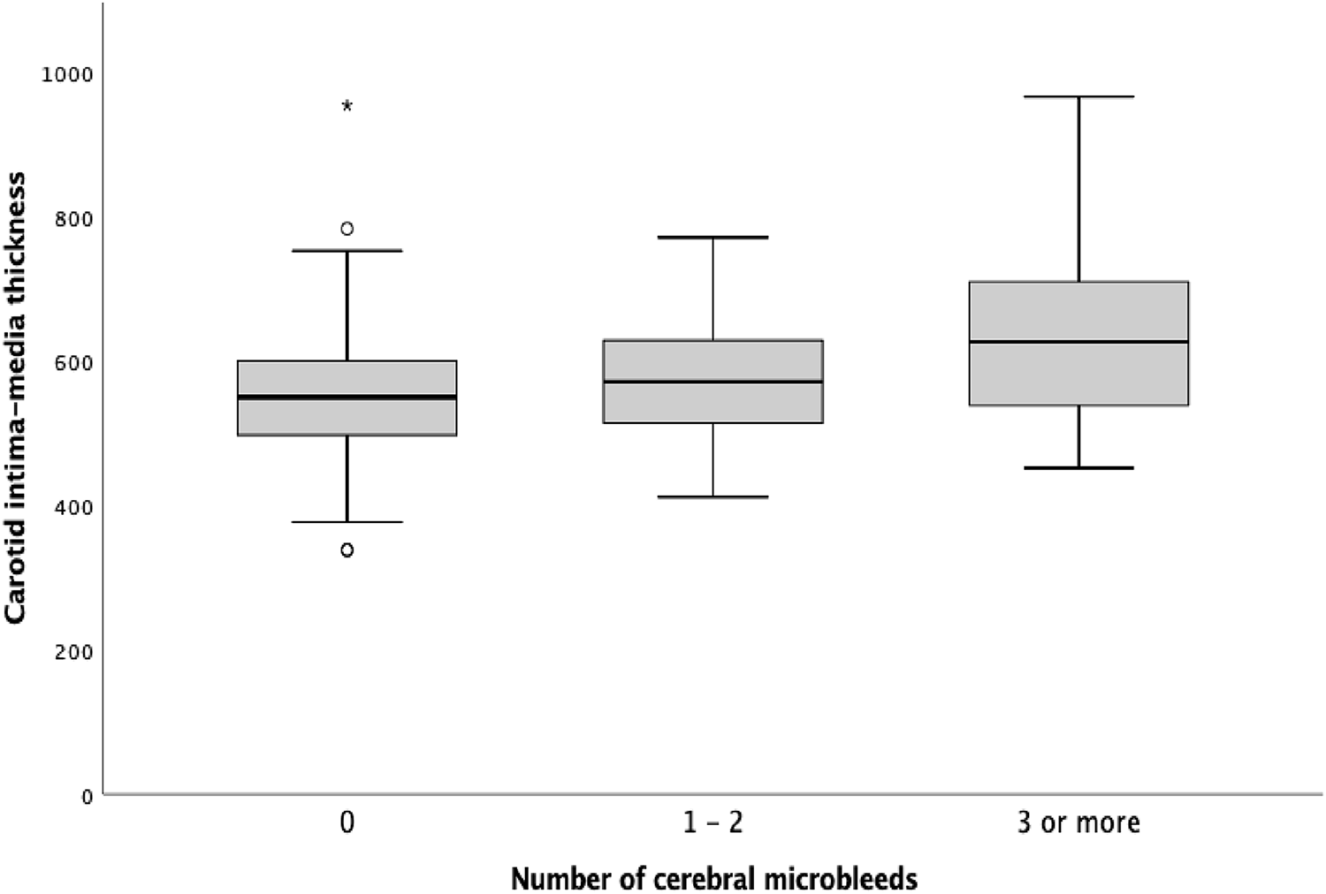
Carotid intima-media thickness (μm) by number of cerebral microbleeds in individuals with type 1 diabetes. Individuals with type 1 diabetes subdivided into three groups based on the number of cerebral microbleeds (zero [*n* = 142], one to two [*n* = 32], more than two [*n* = 12]) with a CIMT of 556 (502–607) μm vs. 578 (513–640) μm vs. 634 (533–737) μm, *p* = 0.028, respectively

In individuals with type 1 diabetes, those with cSVD had an increased CIMT compared to those without (582 [537–653] μm vs. 552 [496–601] μm, *p* = 0.002), as shown in Table 2. Similarly, CIMT was higher in individuals with CMBs (583 [525 – 663] μm) compared to those without (556 [502—607] μm, *p* = 0.016) as well as in those with WMHs (580 [549–654] μm) compared to those with no signs of WMHs (563 [498–613] μm, *p* = 0.019) in brain MRIs. Furthermore, we observed a positive correlation between CIMT and the number of CMBs (Fig. 1).

**Table 2:** Carotid intima-media thickness and arterial stiffness stratified by small-vessel disease findings in brain MRI in individuals with type 1 diabetes (n = 186)

	cSVD			CMBs			WMHs		
	Presence (*n* = 64)	Absence (*n* = 122)	*p*	Presence (*n* = 44)	Absence (*n* = 142)	*p*	Presence (*n* = 31)	Absence (*n* = 155)	*p*
CIMT, μm, median (IQR)	582 (537–653)	552 (496–601)	0.002	583 (525–663)	556 (502–607)	0.016	580 (549–654)	563 (498–613)	0.019
Central PWV, m/s, median (IQR)	7.9 (6.4–9.6)	7.0 (6.1–8.1)	0.018	7.7 (6.2–9.8)	7.3 (6.1–8.3)	0.117	8.3 (7.2–9.6)	7.1 (6.1–8.5)	0.013
Brachial PWV, m/s, median (IQR)	7.5 (6.8–8.7)	7.5 (6.8–8.4)	0.833	7.6 (6.8–8.8)	7.5 (6.7–8.4)	0.518	7.7 (6.3–8.7)	7.5 (6.8–8.4)	0.794
AIx, %, median (IQR)	18.7 (11.6–23.4)	13.5 (5.7–19.3)	0.006	19.0 (11.3–26.3)	14.3 (6.4–20.6)	0.015	19.6 (14.2–23.9)	14.3 (5.4–21.6)	0.006

CIMT = carotid intima-media thickness, PWV = pulse wave velocity, AIx = augmentation index, cSVD = cerebral small-vessel disease, CMBs = cerebral microbleeds, WMHs = white matter hyperintensities

Bivariate correlations between CIMT and the number of CMBs and WMHs in individuals with type 1 diabetes are presented in Supplementary Table 1. Notably, the number of both CMBs (*R* = 0.157, *p* = 0.032) and WMHs (*R* = 0.203, *p* = 0.005) showed significant correlations with CIMT. Lastly, we observed an independent association between CIMT and CMBs in each of the four multivariable models (Model 1: 1.006 [1.001–1.010], *p* = 0.015, Model 2: 1.005 [1.000–1.010], *p* = 0.046, Model 3: 1.005 [1.001–1.009], *p* = 0.024, Model 4: 1.005 [1.001–1.009], *p* = 0.026) adjusted for clinically relevant risk factors (Table 3).

**Table 3:** Multivariable logistic regression analysis of individuals with type 1 diabetes with A) cSVD, B) CMBs and C) WMHs as dependent variables

	**CIMT**	**AIx**	**Central PWV**
	OR (95% Cl); *p* value	OR (95% Cl); *p* value	OR (95% Cl); *p* value
**A) cSVD**			
Model 1	1.004 (0.999–1.008);	1.006 (0.967–1.046);	1.075 (0.842–1.372);
	*p* = 0.086	*p* = 0.778	*p* = 0.563
Model 2	1.003 (0.999–1.008);	1.010 (0.974–1.048);	1.029 (0.794–1.333);
	*p* = 0.140	*p* = 0.579	*p* = 0.831
Model 3	1.004 (1.000–1.008);	1.011 (0.974–1.049);	1.085 (0.863–1.364);
	*p* = 0.057	*p* = 0.577	*p* = 0.486
Model 4	1.005 (1.001–1.009);	1.013 (0.973–1.054);	1.113 (0.867–1.429);
	*p* = 0.016	*p* = 0.538	*p* = 0.399
**B) CMBs**			
Model 1	1.006 (1.001–1.010);	1.008 (0.964–1.054);	1.105 (0.846–1.444);
	*p* = 0.015	*p* = 0.718	*p* = 0.463
Model 2	1.005 (1.000–1.010);	1.010 (0.969–1.053);	1.102 (0.827–1.468);
	*p* = 0.046	*p* = 0.637	*p* = 0.506
Model 3	1.005 (1.001–1.009);	1.017 (0.975–1.061);	1.077 (0.844–1.375);
	*p* = 0.024	*p* = 0.433	*p* = 0.550
Model 4	1.005 (1.001–1.009);	1.003 (0.959–1.049);	1.117 (0.847–1.472);
	*p* = 0.026	*p* = 0.893	*p* = 0.434
**C) WMHs**			
Model 1	1.002 (0.997–1.006);	1.032 (0.978–1.089);	1.074 (0.804–1.434);
	*p* = 0.496	*p* = 0.250	*p* = 0.631
Model 2	1.002 (0.997–1.007);	1.049 (0.997–1.104);	1.031 (0.749–1.420);
	*p* = 0.451	*p* = 0.067	*p* = 0.851
Model 3	1.003 (0.998–1.008);	1.038 (0.983–1.095);	1.117 (0.836–1.493);
	*p* = 0.213	*p* = 0.179	*p* = 0.454
Model 4	1.004 (1.000–1.009);	1.052 (0.997–1.110);	1.149 (0.854–1.545);
	*p* = 0.065	*p* = 0.064	*p* = 0.360

Model 1: Adjusted for age, sex, eGFR, ApoB and SBP

Model 2: Adjusted for age, eGFR, ApoB, SBP, albuminuria, history of retinal photocoagulation and HbA_1c_

Model 3: Adjusted for age, sex, eGFR, LDL, BMI, HbA_1c_, statin therapy and antihypertensive medication

Model 4: Adjusted for sex, eGFR, ApoB, SBP, HbA_1c_ and diabetes duration

CIMT = carotid intima-media thickness, PWV = pulse wave velocity, AIx = augmentation index, cSVD = cerebral small vessel disease, CMBs = cerebral microbleeds, WMHs = white matter hyperintensities, SBP = systolic blood pressure, eGFR = estimated glomerular filtration rate, LDL = low-density lipoprotein, ApoB = apolipoprotein B, BMI = body mass index

### Arterial stiffness parameters and cSVD

In individuals with type 1 diabetes, those with cSVD had higher cPWV (*p* = 0.018) and AIx (*p* = 0.006) compared to those without any signs of cSVDs in their brain MRIs. No differences in bPWV were observed between the groups. Specifically, individuals with type 1 diabetes and CMBs had higher AIx (*p* = 0.015) compared to those without CMBs. In contrast, no differences in bPWV or cPWV were observed between these groups. AIx (*p* = 0.006) and cPWV (*p* = 0.013) were elevated in individuals showing signs of WMHs. bPWV did not differ between participants with and without WMHs (Table 2).

Bivariate correlations between arterial stiffness and cSVD changes are presented in Supplementary Table 1. An association was observed between WMHs and cPWV (*p* = 0.012). This correlation was, however, not independent of known cardiovascular risk factors in the multivariable regression analyses (Table 3).

## Discussion

The main finding of this study was the independent association between CIMT and CMBs in young individuals with type 1 diabetes without any clinical signs of neurological disease, indicating a relationship between subclinical systemic atherosclerosis and early-onset microvascular pathology in the brain.

Previous data on the relationship between CIMT and CMBs are conflicting and limited to the general population [16, 17]. In a community-based study, CIMT was associated with the presence of CMBs in a general population older than our study population [16]. In contrast, another study showed no association between baseline CIMT and CMBs [17].

CIMT is a strong predictor of cardiovascular disease [18]. The observed association between CIMT and CMBs supports the assumption that risk factors for cerebral macrovascular disease are similar to those in preclinical hemorrhage-prone cerebral cSVD. CIMT did, however, not correlate independently with WMHs or lacunes in our study. Teasing out the exact risk factors for hemorrhagic and ischemic end events in the brain caused by cSVD is challenging, especially since CMBs are associated with an increased risk of both intracerebral hemorrhage (ICH) and ischemic stroke, reflecting common underlying mechanisms [7]. Multiple lobar CMBs were seen in the individuals with type 1 diabetes in our study. Lobar CMBs are in turn thought to represent cerebral amyloid angiopathy (CAA), which is one of the most common etiologies causing ICH [5].

Arterial stiffness can be estimated non-invasively using surrogate markers, such as carotid or brachial pulse wave velocity (cPWV and bPWV) and augmentation index (AIx), all available by applanation tonometry. PWV, the gold standard of arterial stiffness, is an established risk factor for cardiovascular disease and mortality in the general population as well as several patient groups [19–23]. In community-based studies, higher PWV has repeatedly been associated with WMHs and brain atrophy, whereas associations with CMBs and lacunar infarcts are scarce [24–26]. Accelerated arterial stiffening seen in individuals with type 1 diabetes correlates with vascular complications such as diabetic retinopathy, nephropathy and cardiovascular disease [27]. Arterial structure and function have also been found to be associated with a greater risk of cognitive impairment and greater blood pressure pulsatility reflecting a comprehensive association between pathology in the arterial tree and end-organ damage in these patients [28–30].

In our study, arterial stiffness was increased in those with cSVD compared to those without. The finding was, however, not independent of the well-known risk factors for arterial disease in type 1 diabetes. This observation differs from that in a previous study showing aortic stiffness to be independently associated with WMHs [31]. The contradiction between these results is not fully clear but might be due to differences in age, diabetes duration and the prevalence of WMHs. Mechanisms other than hemodynamic status such as low-grade inflammation and reactive oxygen species known to be related to vascular disease in type 1 diabetes may explain the lack of association [32]. The complexity of the cross-talk between diabetes and arterial function and structure may also involve endothelial dysfunction, this effect being possibly mediated via kidney damage [33, 34]. Diabetes mellitus has been suggested to associate with dysfunction of the blood–brain barrier [35]. Whether or not blood–brain barrier dysfunction relates to cSVD, and especially to CMBs, is an interesting topic and deserves more research.

Our study findings raise intriguing future research topics relating systemic arterial functional and structural changes to the cerebrovasculature and the relation of these finding into a broader perspective of early arterial aging and accelerated by diabetes [36–38].

This study does not go without limitations. Although our study is, to the best of our knowledge, the largest to assess the relationship between vascular structural and functional parameters and cSVD in individuals with type 1 diabetes to date, an even larger study would enable greater statistical power. We observed an association between CIMT, an acknowledged marker of atherosclerosis and strong predictor of CVD, and CMBs that was independent of a number of risk factors for these entities. By studying an even larger cohort, we may have been able to detect associations between arterial functional (arterial stiffness) variables and other cerebrovascular pathology observed in the individuals with type 1 diabetes. The limited number of control subjects in our study can cause a potential type 2 error in statistical analysis. Further, acknowledging the cross-sectional study design, we can only speculate about causal relationships. Follow-up studies are needed to gain insight into the causality of risk factors. The strengths of this study are the standardized imaging and clinical assessment, as well as the strong phenotypic data.

## Conclusion

Our study shows that vascular structural changes, assessed by CIMT, are associated with the presence of CMBs in individuals with type 1 diabetes, independently of relevant clinical covariates. We did not observe an independent association between functional and structural vascular changes and the overall presence of cSVD. The main finding of this study suggests that CIMT, which is readily available in a clinical setting, adds to risk prediction of early preclinical cerebrovascular disease in individuals with type 1 diabetes. This approach, part of a detailed work-up aiming to individualize treatment in individuals with type 1 diabetes, is feasible in various settings. This may be important considering that these patients are frequently managed by different health professionals possibly altering the quality of care. This may be of special interest for the care of individuals in need of antithrombotic medication, another risk factor for hemorrhage in the brain, as well as for offering more aggressive non-medical prophylactic approaches for individuals with subclinical increased risk for vascular disease.

## Supplementary Information

Supplementary material 1 (DOCX 14 kb)

## Data Availability

The database is available for all FinnDiane researchers.
