# Supplementary material for: Carotid intima-media thickness and arterial stiffness in relation to cerebral small vessel disease in neurologically asymptomatic individuals with type 1 diabetes
Source: Acta Diabetol. 2021 Mar 20;58(7):929–37. doi: 10.1007/s00592-021-01678-x (PMC8187193; doi:10.1007/s00592-021-01678-x)
Supplement: Supplementary file 1 — Supplementary material 1 (DOCX 14 kb) [file 592_2021_1678_MOESM1_ESM.docx]

**Supplementary Table 1** Bivariate correlations between carotid intima-media thickness, measurements of arterial stiffness and the number of cerebral microbleeds and white matter hyperintensities in individuals with type 1 diabetes

|  | **CIMT** | **Central PVW** | **AIx** |
| --- | --- | --- | --- |
| **Number of CMBs** | *R* = 0.157;  *p* = 0.032 | *R* = 0.151;  *p* = 0.059 | *R* = 0.105;  *p* = 0.162 |
| **Number of WMHs** | *R* = 0.203;  *p* = 0.005 | *R* = 0.200;  *p* = 0.012 | *R* = 0.112;  *p* = 0.137 |

CIMT = carotid intima-media thickness, PWV = pulse wave velocity, AIx = augmentation index, CMBs = cerebral microbleeds, WMHs = white matter hyperintensities.
